# The role of hormones in renal disease and ischemia-reperfusion injury

**DOI:** 10.22038/ijbms.2019.34037.8095

**Published:** 2019-05

**Authors:** Shokofeh Banaei, Lotfollah Rezagholizadeh

**Affiliations:** 1 Social Determinants of Health (SDH) Research Center, Ardabil University of Medical Sciences, Ardabil, Iran; 2 Department of Physiology, School of Medicine, Ardabil University of Medical Sciences, Ardabil, Iran; 3 Department of Biochemistry, School of Medicine, Ardabil University of Medical Sciences, Ardabil, Iran

**Keywords:** Acute kidney injury Antioxidant, Hormones, Ischemia-reperfusion injury, Renal disease

## Abstract

The patients with renal diseases, especially end-stage renal disease (ESRD), are at high risk of developing cardiovascular disturbances. Some hormones such as brain natriuretic peptide appear to be important serum biomarkers in predicting cardiac death in ESRD patients. Renal diseases cause inflammation, anemia, uremic toxins, fluid overload, and electrolyte disturbance. Kidney transplantation is considered the choice treatment for patients with ESRD. Ischemia-reperfusion (IR), which occurs during renal transplantation is one of the factors that affect the outcome of renal transplantation. Renal graft rejection is the result of IR injury and there is no effective treatment to prevent IR injury. Reperfusion after ischemia may cause injury through generation of reactive oxygen and nitrogen species, inflammatory responses by increased levels of tumor necrosis factor-α (TNF-α) and interleukins (IL), and apoptotic processes, and leads to acute kidney injury (AKI). Thus, antioxidant, anti-apoptotic and anti-inflammatory hormones, which inhibit these pathways, can protect against IR injury and improve transplanted renal function in patients with ESRD.

## Introduction

Acute kidney injury (AKI) is characterized by decreased renal function, exhibited as decreased urine output and increased serum creatinine levels. AKI occurs in patients with acute kidney disease and is an acute complication of cardiac surgery. Several events cause AKI, including obstruction of the urinary tract, exposure to toxins and renal ischemia. AKI may lead to a number of complications, including uremia, body fluid imbalance, and metabolic acidosis (1). 

Renal ischemia-reperfusion occurs in clinical settings, such as renal transplantation for ESRD patients, increases immune activation and antibody production that contribute to the loss of renal grafts and graft dysfunction. Oxygen free radicals are produced during the reperfusion phase, which causes lipid peroxidation and promotes tissue damage. Oxidative damage to DNA and proteins and lipid membrane peroxidation can cause cell death and apoptosis (2, 3). IR injury may decrease antioxidant enzymes such as superoxide dismutase (SOD), catalase (CAT), and glutathione peroxidase (GPx). Reactive oxygen species (ROS) contribute to the pathology of renal IR injury. ROS can oxidize many cell constituents, including proteins, lipids, and DNA and impose a threat to cell cytoskeleton (4). 

Cells have evolved several defense mechanisms to cope with oxidative damage, among which autophagy plays an important role. The specific autophagic processes in response to ROS, including chaperone-mediated autophagy and the degradation of mitochondria, have been suggested to reduce the oxidative injury caused by defective mitochondria. Members of the heat shock protein (HSP) family, such as HSP25 and HSP27 are molecular chaperones involved in enhancing tolerance to oxidative stresses and may possess anti-apoptotic effects (5, 6). ROS such as superoxide and hydrogen peroxide elicited expression changes of multiple genes, for example, microRNAs, single-stranded noncoding RNAs of approximately 22 nucleotides, are responsible for ROS-mediated cell injuries such as necrosis and apoptosis. The expression changes of microRNAs (miRNAs) following ROS stimulation could be critical in ROS-mediated regulations of signaling transduction pathways and gene expression. Dys-regulated miRNA expression has been found to be involved in renal IR injury. However, the synthesized miRNAs have been demonstrated to be protective after IR injury, they are able to be released into circulating blood from ischemic tissues. MiRNAs in the peripheral blood have been reported to be useful biomarkers for diseases such as liver injury and renal ischemia (3).

The patients with renal diseases, especially end-stage renal disease (ESRD), are high risk in developing cardiovascular disturbances. Renal diseases cause inflammation, anemia, uremic toxins, fluid overload, and electrolyte disturbance. The risk of cardiovascular diseases such as ventricular hypertrophy, cardiac ischemia, heart failure, and atherosclerosis is higher in ESRD patients (7). On the other hand, the antioxidant, anti-apoptotic and anti-inflammatory hormones, which inhibit inflammatory and oxidative pathways, can protect against IR injury and improve cardiovascular disturbances and transplanted renal function in patients with ESRD.

**Ghrelin and obestatin**

Malnutrition is a common problem and has undesirable effects on patients with ESRD. The reason for malnutrition is lack of appetite caused by the inflammation and protein loss in dialysis patients. There is a relationship between nutrition regulating hormones and malnutrition in ESRD patients. Ghrelin is a hormone that regulates body weight and eating behavior. Exogenous ghrelin supplementation stimulates food intake and appetite. Ghrelin is a peptide hormone that has 28 amino acids and is secreted by the stomach, also it is expressed by renal cells. The ghrelin level is about 2.8 times higher in ESRD patients. This is due to renal failure to dispose of and destroy ghrelin (8).

In ESRD patients, serum ghrelin levels increase, especially after bilateral nephrectomy. Thus, this indicates that kidneys play a major role in disposing of and destruction of ghrelin. It has been shown that the ghrelin gene is expressed by kidneys and ghrelin receptors are found in tubular and glomerular epithelial renal cells. The levels of ghrelin are low in obese patients and are increased with weight loss. The decrease and insensitivity of ghrelin receptors might be arisen by increased ghrelin levels in ESRD patients. On the other hand, the post-hemodialysis ghrelin levels are lower than pre-hemodialysis ghrelin levels. This indicates that serum ghrelin is cleared by hemodialysis. Also, in ESRD patients saliva ghrelin levels are increased and are higher in pre-hemodialysis (5).

Obestatin, a peptide hormone, derived from pro-ghrelin, is synthesized by the stomach and small intestine cells. Obestatin and ghrelin might be involved in anorexia in ESRD patients. The obestatin hormone is encoded by the same gene as ghrelin and has the opposite effects compared with ghrelin, it inhibits nutrition and weight gain, whereas ghrelin stimulates nutrition. Obese women have lower serum obestatin levels due to elevation in the ghrelin-obestatin ratio so that, it seems that, these hormones may be associated with the regulation of body weight. The balance between obestatin and ghrelin levels in ESRD patients affects energy balance and appetite, and this contributes to malnutrition in ESRD patients. Thus, obestatin is a nutrition marker indicating insulin resistance and fattening of the body (9).

Obestatin prevents ischemia-reperfusion-induced apoptosis, both ghrelin and obestatin exert potent anti-apoptotic, anti-inflammatory, and antioxidant effects in IR injury. Administered ghrelin before reperfusion reduces serum levels of interleukins and TNF-α. Thus, ghrelin decreases the inflammatory response to IR injury and protects against renal IR injury. In addition, ghrelin can upregulate the PI3K/AKT signaling pathway by enhancing the microRNA-21(miR-21) levels and ameliorate IR-induced AKI by upregulating miR-21, which leads to the protective effects of ghrelin on renal IR injury by inhibiting kidney tubular epithelial apoptosis and inflammatory responses (10). Similar to ghrelin, obestatin exerts protection in renal IR injury via its anti-inflammatory and anti-apoptotic effects. Also, obestatin can decrease oxidative damage and ameliorate renal ischemia-reperfusion injury (6). 

**Aldosterone**

Kidney injury molecule (KIM) is a glycoprotein that is usually not detected in the healthy kidney, but is excreted in the urine during ischemic-induced tubule injury. Kim is a biomarker of the proteinuric and ischemia-associated renal diseases. Kim has been detected due to its pivotal role in the regulation of tubular injury and repair. Studies have demonstrated that KIM may be associated with the proliferation of proximal tubule cells and may participate in maintaining their functional integrity (1).

Aldosterone (Aldo) is a chief mediator of the renin-angiotensin-aldosterone system (RAAS) and exerts an important role in the modulation of extracellular fluid and salt. Also, Aldo has non-hemodynamic effects involved with renal damage. It has been found that RAAS-induced KIM expression in the proximal tubule, leads to renal damage, whereas pretreatment with spironolactone, the Aldo receptor antagonist, is able to reduce KIM expression and attenuate renal injury. Thus Aldo has a pivotal role in the KIM expression in renal tissues during AKI. The Aldo levels in the renal tissues, plasma, and urine are increased in the AKI. It has been reported that spironolactone exerts renal protective effects by reduction of KIM expression and attenuates renal injury (1).

Diabetic nephrology is the cause of renal failure leading to ESRD in patients. There are albuminuria and expansion of the glomerular and tubular mesangial matrix in this nephropathy. Studies have demonstrated that inhibitors of Aldo cause a decrease in the progression of diabetic nephropathy and renal fibrosis, also, these studies have suggested that Aldo receptor blockers, Aldo antagonists, ameliorate diabetic kidney complications. Thus, the mineralocorticoid receptor blockers have renoprotective properties through decreased diabetic renal complications such as proteinuria, hypertension, and glomerulopathy (8).

Spironolactone, an Aldo antagonist, ameliorates the progression of sclerosis and fibrosis in nephritis, also the presence of mineralocorticoid receptors on fibroblasts, podocytes, and mesangial cells confirms the anti-fibrotic effects of spironolactone. However, the vasoconstrictor actions of Aldo are not affected by spironolactone. Therefore, Aldo increases mesangial cell proliferation, hypertension, and inflammation, and Aldo blockade ameliorates inflammation, glomerular hypertrophy and albuminuria of diabetic renal complications (11). The role of RAAS in water and sodium retention to regulate electrolyte-fluid balance and blood pressure is well established. RAAS contributes to the pathophysiology of renal disease; the activities of Aldo and angiotensin are fundamental to the pathological and physiological effects of the system and is a key component for the management of chronic kidney disease (CKD) and hypertension (12).

**Sex hormones**

Other factors, such as sex and growth hormones contribute to the development of diabetic nephropathy in ESRD patients. Testosterone, a male sex hormone, has been suggested to be a risk factor for the progression of diabetic nephropathy, it exacerbates renal disease, while estrogens slow the progression rate of renal disease. The absence of testosterone decreases the progression of renal disease (13). Hypertensive renal disease is common in men compared with women, and premenopausal females are less susceptible than men to heart disease and hypertensive kidney disease. However, after menopause, the incidence of kidney disease in females elevates suggesting that the loss of sex hormones contributes to the development of renal disease. 17 b-estradiol decreases age-related kidney dysfunction; hypertensive renal disease, and nephron-sclerosis are more common post-menopause compared with pre-menopause in women. The results confirm the protective role of ovarian hormones on hypertensive renal disease, and estrogen or progesterone replacement therapy improves kidney function. Also, these data demonstrate the involvement of genetic mechanisms in hypertensive renal disease in both pre-menopausal and post-menopausal states (14). 

Low dose testosterone protects against renal IR by reducing pro-inflammatory cytokine production and promoting anti-inflammatory cytokine synthesis. The decreased infiltration of leukocytes by testosterone can lead to less inflammation and oxidative stress in renal tissue. Also, testosterone can cause renal vasodilation in the renal medulla after IR. Testosterone is a vasodilator and treatment with vasodilators has been demonstrated to protect against renal IR. It causes a decrease in afferent resistance in kidneys by increasing medullary perfusion and causes renal protection, as well as inflammation reduction. Also, testosterone infusion can play a pivotal role in decreasing plasma creatinine after renal IR. The inhibition of calcium channels and NO release are the mechanisms by which testosterone causes vasodilation in vascular beds. Therefore low dose testosterone, by increasing NO and decreasing inflammation, can protect after renal IR (15). 

**Parathyroid hormone**

In end-stage renal disease or in patients with chronic kidney disease, circulating levels of 1, 25 dihydroxy-vitamin D (1, 25 (OH) _2_ D) are reduced. It is due to decreased synthesis of 1, 25(OH)_2_D by the kidney and decreased conversion of 25-hydroxyvitamin D to 1, 25 (OH)_2_ D by the poorly functioning kidney. Also, diminished glomerular filtration rate in chronic kidney disease causes decreased 25-hydroxyvitamin D (25(OH)D) delivery to the kidney, which contributes to decreased 1, 25 (OH)_2_ D levels. On the other hand, the reduction of 1, 25 (OH)_2_ D production by the kidney impairs gastrointestinal calcium (Ca^2+^) absorption and stimulates parathyroid hormone (PTH) secretion. Then, PTH increases phosphate and Ca^2+ ^ efflux from bones and stimulates renal 1, 25 (OH)_2_ D production , eventually, the renal calcium reabsorption is increased and renal phosphate reabsorption is inhibited, until compensatory mechanisms become insufficient, elevated PTH levels maintain the serum levels of calcium and phosphate in the normal range, therefore, parathyroid gland hypertrophy occurs (16). Sleep disturbance is one of the most important problems in patients with chronic renal disease on hemodialysis. Its pathophysiology and the role of contributing hormones and factors are not understood. It has been postulated that bad sleep quality is associated with high parathyroid hormone levels in hemodialysis patients (17).

CKD is associated with bone and mineral disorders including abnormalities of phosphorus (P), calcium (Ca), vitamin D, parathyroid hormone, and vascular calcification. Vascular calcification is associated with renal ischemia, it occurs by phosphorus and calcium precipitation and is produced by a process in vascular smooth muscle cells undergoing apoptosis. Also, matrix formation and attraction of local factors are involved in the mineralization process and in the pathogenesis of vascular calcification. CKD patients have a high prevalence of vascular calcification, because of cardiovascular disorders, hypertension, dyslipidemia, hyperphosphatemia, and hyperparathyroidism. Patients with CKD develop hyperphosphatemia due to impaired renal excretion. High serum phosphorus and calcium levels are considered vascular toxins that play a pivotal role in the development of vascular calcification (18).

Hyperparathyroidism occurs in the early stage of CKD and progression of CKD causes hypocalcemia and hyperphosphatemia that lead to hyperparathyroidism and parathyroid gland hyperplasia. High PTH levels are responsible for the increased bone resorption in CKD. A severe reduction in serum 1, 25 dihydroxy vitamin D level occurs in CKD. Low 25 hydroxyvitamin D levels affect mortality and survival in CKD patients. 1, 25 dihydroxy vitamin D has been demonstrated to act as a negative regulator of RAAS, which plays a critical role in electrolyte homeostasis and modulating volume. The administration of physiological doses of 1, 25 dihydroxy vitamin D has been reported to protect against vascular calcification in CKD patients (18).

**Figure 1:**
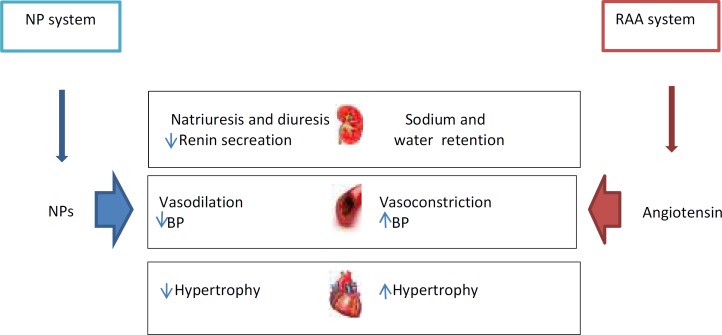
A diagram to show how the NP system and the RAA system interact in order to maintain cardio-renal homeostasis

BP: blood pressure; NP: natriuretic peptide; RAA: renin-angiotensin-aldosterone

**Natriuretic peptides**

Renal diseases such as chronic kidney disease and hypertension are associated with mortality. Natriuretic peptides (NPs) are cardiovascular hormones that affect neuro-hormonal activity, vascular tone, and cardiovascular remodeling. The beneficial physiological effects of NPs are used for treatment of renal diseases. NPs are paracrine and endocrine peptides, including atrial natriuretic peptide (ANP), B-natriuretic peptide (BNP), and C-natriuretic peptide (CNP). ANP is released in response to atrial distension, BNP is secreted by ventricular myocytes especially in pathological states, and CNP is secreted by the vascular endothelium by inflammatory cytokines. It has been found that ANP is the physiological hormone of NPs, as it controls renal functions, in contrast, BNP functions in pathological states as a cardiac stress response peptide. BNP and CNP circulating levels are low in the absence of pathological disease states. NPs play a notable role in the control of intravascular volume and blood pressure (BP) (19). 

They regulate BP by suppressing RAAS and decreasing sympathetic tone. Also, NPs play a pivotal role in the regulation of fluid and electrolyte balance in the kidney. They inhibit sodium reabsorption in the nephrons, which leads to the promotion of diuresis and natriuresis and reduces BP. These effects of NPs on fluid-electrolyte balance are mediated more by ANP than BNP and CNP in the kidney. ANP increases glomerular filtration rate and renal blood flow. Neuro-hormonal systems such as the sympathetic nervous system (SNS), RAAS, and the NP system play a pivotal role in regulating fluid and electrolyte modulation and vascular tone. The interaction between NPs and SNS and RAAS maintains the cardiorenal homeostasis. Indeed, the physiological activity of the NPs is counter or antagonist with the two systems RAAS and SNS, this means that the NPs suppress RAAS, inhibit SNS effects and increase parasympathetic tone. SNS enhances renin secretion from the kidneys, thereby increases RAAS activity, SNS and RAAS act in a cooperative manner, as a result, both of the two systems reduce secretion of ANP (12).

ANP is involved in the pathogenesis of hypertension, mainly due to the important role of the kidney in the development of hypertension, and effects of ANP on diuresis, natriuresis, and hypertension. The administration of ANP causes diuresis and natriuresis in animal models. On the other hand, decreased production of ANP leads to hypertension. In patients with essential hypertension, increasing plasma levels of ANP increase salt excretion and suppress the RAA system. Thus, ANP plays an essential role in renal homeostasis in patients with hypertension. Also, hypertension may be the result of a deficiency in biologically active natriuretic peptides (12).

Brain natriuretic peptide (BNP) is a peptide hormone released by the ventricular myocytes in response to increased ventricular filling pressure and stretch. The BNP level is a useful screening test for heart failure in ESRD patients; there is valuable evidence that BNP is an important prognostic tool in ESRD patients. In hemodialysis or peritoneal dialysis patients, BNP is associated with ventricular hypertrophy and cardiac dysfunction. Studies have demonstrated that elevated BNP is a valuable biomarker in diagnosing ventricular hypertrophy in dialysis patients. Therefore, BNP has clinical implications and BNP measurement may be used to identify ESRD patients at risk of cardiac death. It appears to be an important serum biomarker in predicting cardiac death in ESRD patients (20).

The morbidity and mortality after renal transplantation are influenced by IR injury. Thus, agents such as ANP and CNP protecting against IR injury are very important. The protection of the kidney against ischemic injury by ANP has been reported. ANP is a vasodilator hormone, its vasodilating properties were thought to be involved in the protection against renal ischemic injury. Several experiments have revealed ANP effect on the immune function. ANP inhibits activation of nuclear factor ҡB (NF-ҡB) during the reperfusion phase. NF-ҡB has an important role among the intracellular events occurring during reperfusion. The activation of NF-ҡB is stimulated by different factors such as ROS, endotoxin, and reperfusion upon renal transplantation for ESRD patients. NF-ҡB initiates the transcription of genes that are involved in inflammatory pathways including interleukins and TNF-α. Therefore, NF-ҡB can be a pivotal transcription factor involved in IR injury. The findings show that augmentation of NF-ҡB is important for renal IR injury and ANP protects the kidney via influence on NF-ҡB. Also, ANP-signaling can be a hormonal defense mechanism to protect the kidney against the toxic effects of NF-ҡB. The attenuation of NF-ҡB activation protects the kidney from IR injury by ANP (21). 

The plasma levels of NPs are increased in patients with chronic kidney disease. In a study of patients with CKD, plasma levels of BNP increased, renal function decreased and was associated with raised risk for progression of CKD to ESRD (20). It was found that pro BNP is a predictor of CKD and it may be an important marker of prognosis CKD progression. Also, a recent study indicated that increased BNP is a predictor of CKD progression to ESRD (22). Therefore, the assessment of plasma levels is a valuable method to recognize higher cardiovascular risk in patients with CKD and the utilization of natriuretic peptides is a therapeutic strategy for the treatment of renal diseases. The increasing NP levels can be achieved by the administration of exogenous natriuretic peptides (Figure 1) (12).

**Cortisol**

Cortisol is secreted following stress, follows the circadian rhythm, and circulating cortisol levels are low at night and at peak in the morning. Cortisol secretion is reduced in patients with kidney impairment and the infusion of exogenous cortisol causes a rise in glomerular filtration rate, natriuresis, and diuresis by the increase in renal blood flow. An increase in filtered phosphate and a fall in phosphate reabsorption increase urinary phosphate excretion. Cortisol enhances the capacity of the kidney to regulate fluid and electrolyte homeostasis by raising GFR and delivering more water and sodium to the distal nephron where the reabsorption of water and sodium is regulated in accordance with need (23). 

The salivary cortisol level is essentially correlated with plasma cortisol in normal kidney function. End-stage renal disease subjects have a rise in plasma and salivary cortisol levels, the ESRD patients have a reduced sensitivity to glucocorticoid negative feedback. So, the decreased glucocorticoid feedback sensitivity leads to a rise in plasma adrenocorticotropic (ACTH). It is clear that the relationship between plasma cortisol and ACTH is intact in ESRD patients, but normal cortisol circadian rhythm is disrupted in ESRD patients; it may be associated with changes in the characteristics of ESRD subjects. Subjects with CKD have increased markers of inflammation that are related with abnormalities such as resistance to erythropoiesis mediated-agents; an increase in plasma c-reactive protein (CRP) causes the disruption of cortisol circadian rhythm in ESRD patients. Thus, it seems that ESRD patients with raised inflammatory agents may have a high potential for a disrupted circadian rhythm and reduced sensitivity to cortisol feedback (24).

In patients with ESRD undergoing dialysis, hypotension is related to high mortality. In patients with chronic renal failure (CRF), hypotension is a key marker of adrenal insufficiency. Deficient adrenal secretion is diagnosed when salivary Aldo and cortisol are released after stimulation with ACTH. Steroid replacement improves BP in the ESRD with adrenal insufficiency, taking glucocorticoids are successful to achieve normal adrenal function (25).

Inflammation contributes to the pathophysiology of IR damage. Reperfusion after ischemia upon renal transplantation causes the initiation of inflammatory pathways including the release of TNF-α, various interleukins, prostaglandins, and adhesion molecules. Increased inflammatory cytokines circulation rejects the renal graft. Cortisol has two basic anti-inflammatory effects: (1) it can block the early stages of the inflammation process before inflammation even begins, or (2) if inflammation has already begun, it causes rapid resolution of the inflammation and increases the rapidity of healing. Also, cortisol has the following effects in preventing inflammation: cortisol stabilizes the lysosomal membranes, decreases the permeability of the capillaries, decreases migration of white blood cells into the inflamed area, suppresses the immune system and reduces the release of interleukins from the white blood cells. Thus, cortisol can protect the renal tissue against IR damage by its anti-inflammatory properties and improve AKI in ESRD patients (25). 

**Melatonin**

Melatonin (N- acetyl-5-methoxytryptamine) is produced by the pineal gland that functions as a regulator of sleep, circadian rhythm, and immune function. Also, Melatonin (MEL) is a potent ROS scavenger because of its capacity to act as an electron donor. MEL and its metabolites have potent anti-inflammatory and antioxidant properties. MEL not only neutralizes reactive nitrogen species (RNS) and reactive oxygen species (ROS) but also acts through the stimulation of antioxidant enzymatic systems and stabilizing cell membranes (26-28). It activates the gene expression of protective enzymes and decreases apoptosis and lipid peroxidation (29). Thus, MEL can provide effective protection against IR injury during renal transplantation in ESRD patients by scavenging free oxygen radicals and can improve grafted renal function.

Patients with ESRD suffer from mental depression, sleep disorders, and disorders of the endocrine system such as hypothalamic-pituitary axis depression, gonadal dysfunction, and insulin resistance. MEL is metabolized by liver enzymes and excreted by the kidney. In addition, the alterations in MEL rhythm may be associated with the physiology of the hypothalamic-pituitary axis. The elevation of melatonin levels in ESRD patients ameliorates insomnia. Also, the supra-physiological concentrations of MEL in these patients are associated with hypogonadism amenorrhea due to the suppression of the hypothalamus-pituitary axis (30).

**Erythropoietin**

Anemia is due to the impairment of the renal endocrine role in ESRD patients, the renal secretion of erythropoietin (EPO) causes red blood cell (RBC) proliferation in a healthy human, but EPO is insufficiently produced in ESRD patients, which leads to decreased RBC production or hemoglobin. Renal function declines and cardiac disease occurs in anemic CKD patients, thus, the treatment of anemia is critical for the quality of life of CKD patients, and managing anemia will improve the clinical outcomes of these patients. Therefore, the erythropoietin stimulating agents are administrated in CKD-associated anemia. The increased levels of inflammatory cytokines in CKD negatively modulate erythropoiesis, exacerbate the anemic state, and contribute to the EPO responses (31).

EPO, a glycoprotein, is produced by the kidney. Recombinant human EPO (rHuEPO) has been used to treat anemia in clinic, and EPO induces cytoprotection via its receptor expressed in tissue against IR injury in different organs such as the lungs, liver, heart, and kidneys. Endogenous erythropoietin is secreted by renal fibroblasts, and EPO receptors are expressed on endothelial and mesangial cells. EPO pretreatment before ischemia reduces tubular damage and improves renal function (32). In addition, EPO enhances expression of the Bcl2 protein and decreases tubular apoptosis and caspase-3 activation. Caspase-3 has an important role in apoptosis and inflammation in ischemic tissues. Thus, EPO plays the anti-apoptotic and anti-inflammatory effects and decreases inflammatory cytokines. Also, EPO can provide effective protection against IR injury during renal transplantation in ESRD patients with scavenger of free oxygen radicals and can improve grafted renal function (5). The pro-inflammatory cytokines including interleukins, interferon, and tumor necrosis factor contribute to the responsiveness to erythropoietin and these cytokines are associated with poor EPO sensitivity and can affect the process of erythropoiesis in CKD. Thus, EPO therapy can improve anemia in CKD and decrease cardiovascular disease risk (31).

Iron deficiency, inflammation, and decreased EPO production cause anemia in ESRD, and EPO therapy leads to the alleviation of the adverse effects of anemia. However, there is a variation in individual response to EPO that is known as EPO resistance. It is the failure to maintain the desired range of hemoglobin levels despite the administration of standard EPO doses. It seems that EPO resistance is associated with the accumulation of the uremic toxin, inflammatory cytokines, and vitamin deficiencies (33). 

The antioxidant effects of EPO may alleviate oxidative stress in uremic patients in CKD and decrease the EPO resistance mediated by uremic toxins in hemodialysis patients. The findings have approved EPO for the treatment of anemia associated with CKD and it has been confirmed that EPO elevates and sustains hemoglobin levels to decrease the need for RBC transfusions in ESRD patients. Erythropoiesis-stimulating agents cause higher hemoglobin levels and cardiovascular safety in hemodialysis patients. The higher hemoglobin levels improve the quality of life in ESRD patients. On the other hand, the reduction of hemoglobin levels, for example, 13.5 g/dl in CKD patients, was associated with elevated cardiovascular disease and no increased improvement in the quality of life. Of course, the EPO doses should be regulated to prevent EPO resistance in patients. Thus, the regulation of EPO doses is an advantage for clinical picture of CKD patients (34).

**Insulin**

Inflammation contributes to the pathophysiology of IR injury. Reperfusion after ischemia in renal transplantation causes the generation of pro-inflammatory cytokines including myeloperoxidase (MPO) and TNF-α and the increase of circulating inflammatory cytokines rejects the renal graft. Insulin has multiple protective effects including antioxidant and anti-inflammatory, which can protect the kidney from IR injury. Insulin can decrease the generation of TNF-α and MPO and reduce the expression of pro-inflammatory cytokines in ischemic tissue and protect the renal tissue against IR injury and improve AKI in ESRD patients (35). 

The patients with ESRD exhibit pathophysiological alterations such as insulin resistance and hyperglucagonemia. These patients have elevated plasma glucagon levels that are non-suppressible after glucose administration. On the other hand, insulin responses are decreased in all ESRD patients and fasting concentrations of glucagon are increased in these patients, since progression of renal disease is involved with metabolic dysregulation such as impairments in the glucose metabolism and increased risk of diabetes, as a result, the beta cells in the patients with ESRD are unable to respond to increased glucose by insulin secretion.

The findings show glucagon secretion is increased in the hemodialysis patients during meal stimulation and this hyperglucagonemia plays a role in the disturbed glucose metabolism in ESRD patients, also pancreatic alpha and beta cell disturbances may be involved in the pathogenesis of the disturbed glucose metabolism in these patients and these disturbances can be explained by severe uremia (36).

**Insulin-like growth factors**

Insulin-like growth factors (IGFs) are expressed by tubule cell types and secreted by proximal tubule cells. Additionally, some IGFs are early AKI biomarkers. In renal IR, the secretion of IGFs is enhanced after reperfusion and this demonstrates their potential role in the pathogenesis of AKI. IGFs, especially IGF7, are novel biomarkers for the determination of AKI degree in ESRD patients. These biomarkers have been related to predicting renal recovery after AKI and prediction of recovery or dialysis after renal transplantation. Thus IGFs may serve as alarm signals that may be effective in conditions such as acute or chronic kidney injury in ESRD patients. The expression of various IGFs has been reported in the glomerulus and in proximal and distal tubule cells, as a result of the renal response to stress or injury (37).

These findings may demonstrate the value of these biomarkers in etiology and the molecular basis of AKI in ESRD patients. IGFs have been associated with various biological processes such as tumor suppression as a growth suppressor and angiogenesis. It has been hypothesized that the increased secretion of IGFs in AKI may act in renal tubule epithelial cells to prevent the proliferation of injured cells. It has been demonstrated that IGFs, AKI urine biomarkers, secreted and expressed in renal epithelial cells of proximal and distal tubule, serve as potential biomarkers in the protection or recovery from AKI (37, 38). 

## Conclusion

Renal ischemia-reperfusion, which occurs during partial nephrectomy and renal transplantation, which is considered a choice treatment for patients with end-stage renal disease, is a common cause of acute renal failure. Ischemia insult, during renal transplantation, is responsible for graft dysfunction and loss of renal grafts. Reperfusion after ischemia initiates a series of cellular events that lead to apoptotic cell death. Several mechanisms are involved in ischemia-reperfusion pathophysiology such as ATP depletion, oxygen free radicals, and infiltration of inflammatory cytokines. The generation of reactive oxygen species, inflammatory mediators, and the activation of necrotic and apoptotic processes especially in the reperfusion phase result in the lipid peroxidation of the cellular membranes and oxidative DNA damage with the production of toxic metabolites that cause the rejection of transplanted kidney in ESRD patients. Therefore, anti-inflammatory and anti-oxidative hormones such as erythropoietin, melatonin, insulin, etc., which prevent these pathways, can protect against IR damage and improve transplanted renal function and influence the outcome after kidney transplantation in ESRD patients.
